# Developing a generalized nonlinear mixed-effects biomass model at stand-level under different age conditions for Chinese fir based on LiDAR and ground survey data in southern China

**DOI:** 10.3389/fpls.2025.1532138

**Published:** 2025-05-08

**Authors:** Xinsheng Zhu, Tianbao Huang, Ziyang Liu, Lang Bai, Yongfeng Yang, Jinsheng Ye, Qiulai Wang, Ram P. Sharma, Liyong Fu

**Affiliations:** ^1^ Research Institute of Forest Resource Information Techniques, Chinese Academy of Forestry, Beijing, China; ^2^ Hanan Sanya Urban Ecosystem Observation and Research, Staton, Academy of Inventory and Planning, National Forestry and Grassland Administration, Beijing, China; ^3^ Guangdong Forestry Survey and Planning Institute, Guangzhou, China; ^4^ Institute of Forestry, Tribhuvan University, Kathmandu, Nepal

**Keywords:** airborne LiDAR, forest biomass, regression modeling, age grouping, dummy variable modeling

## Abstract

**Introduction:**

Chinese fir *(Cunninghamia lanceolata)* is a crucial afforestation and timber species in southern China. Accurate estimation of its stand biomass is vital for forest resource assessment, ecological industry development, and ecosystem management. However, traditional biomass prediction methods often face limitations in terms of accuracy and efficiency, highlighting the need for more robust modeling approaches.

**Methods:**

This study utilized data from 154 forest stands in Guangdong Province to develop biomass regression models that incorporate random effects and dummy variables. The models were based on airborne LiDAR-derived metrics. Among 41 highly correlated LiDAR variables, only two—5% cumulative height percentile and leaf area index—were retained in the final model.

**Results:**

The results revealed that the logistic mixed-effects model was the most effective for estimating leaf biomass, while the empirical mixed-effects model was better suited for other biomass components. Nonlinear models outperformed linear models, with the nonlinear mixed-effects model (incorporating stand age as a random effect) achieving the highest predictive accuracy. Furthermore, machine learning techniques further improved model performance (R² = 0.855 to 0.939). Validation with independent test samples confirmed the robustness and reliability of the nonlinear mixed-effects model.

**Discussion:**

This study highlights the effectiveness of airborne LiDAR data in providing efficient and precise estimates of stand biomass. It also emphasizes the significant role of stand developmental stages in biomass modeling. The findings contribute to the development of a rigorous and scalable framework for large-scale artificial forest biomass estimation, which has important implications for forest resource monitoring, ecological industry development, and ecosystem management strategies.

## 1 Introduction

As the primary component of terrestrial ecosystems, forest ecosystems exhibit high productivity, biomass, and biodiversity, playing a crucial and irreplaceable role in global ecosystems as well as in human economic and social development (Song et al., 2011; Liu et al., 2015; Organization, A, 2017). Biomass serves as the foundational energy source and nutrient reservoir for the functioning of forest ecosystems, making it one of the most fundamental indicators of their health (Zhao et al., 2016). Additionally, biomass is a significant contributor to the stability of terrestrial ecosystems. Accurately estimating forest biomass and quantitatively analyzing forestry and forest system issues is essential for evaluating the health status of forest ecosystems and for studying forest carbon cycling (He et al., 2013; Stovall et al., 2017). Cunninghamia lanceolata has the advantages of strong adaptability, barren tolerance, rapid growth, low afforestation cost and easy management. It is an important commercial timber tree species in the subtropical region of southern China. The planting area accounts for about 20% of the total amount of China’s plantations, and plays an important role in wood production and forest carbon sequestration (Zhou et al., 2021; Guo et al., 2022). Accurately estimating its biomass is of great significance for forest resource assessment, ecological industry development, and refined forestry management.

Traditional ground survey methods for estimating forest biomass are constrained by human factors, making them inefficient, time-consuming, and incapable of collecting data on a regional or larger scale (Di et al., 2016; Mutwiri et al., 2017). In contrast, the rapid advancement of remote sensing technology allows for the swift acquisition of large-scale, high-temporal data, significantly enhancing forest resource monitoring capabilities (Eisfelder et al., 2012). Optical remote sensing data can quickly and accurately capture large-scale forest growth factors and ecological information, providing valuable support for forest resource management. However, optical remote sensing is limited to obtaining horizontal structural information of forests and does not provide access to three-dimensional structural data (Mao et al., 2022). Compared to traditional field surveying and optical remote sensing technology, LiDAR (Light Detection and Ranging) uses high-frequency laser pulses actively directed at the target to directly obtain precise three-dimensional spatial coordinates and echo information of forest trees, offering advantages such as accurate positioning, high penetration rate, and direct measurement of height. This is beneficial for determining vegetation structure characteristics and estimating biomass at both individual tree and stand scales (Tang et al., 2012; Beland et al., 2019). Currently, LiDAR technology is divided into three types based on the sensor platform: satellite-based, airborne, and ground-based. Airborne LiDAR, due to its low cost, high timeliness, high spatiotemporal resolution, and high mobility, has garnered more attention in forest resource surveys (Maltamo et al., 2004; Noordermeer et al., 2018). In recent years, some European and American countries have begun using airborne LiDAR for large-scale forest resource surveys (Pawe et al., 2017). Studies have shown that it significantly improves the accuracy of forest structural parameter extraction and achieves reliable performance in biomass estimation across various forest types (Pawe et al., 2017; Zhang et al., 2024).

Establishing reliable and accurate biomass inversion models, grounded in the strong correlation between forest biomass and structural parameters, is essential for the effective application of airborne LiDAR technology in forest resource surveys. Currently, biomass inversion primarily relies on regression models, which can be categorized into parametric and non-parametric models. Parametric models are mostly linear regressions (LR), Logistic regressions, etc., while non-parametric models include Support Vector Machines, Random Forests (RF), and more (Zhu et al., 2020; Emilien et al., 2021; Zeng et al., 2021; Xie et al., 2023). Many researchers have analyzed data based on radar variables and stand parameters, achieving good inversion results in biomass estimation for different forest types within specific research areas. The variables extracted from airborne LiDAR data mainly consist of height characteristics and canopy characteristics (Lim and Treitz, 2004). Wallace et al. used UAV LiDAR systems for forest stand structure assessment, exploring the potential of UAVs in measuring and monitoring forest structural characteristics (Wallace et al., 2016). Xie et al. analyzed the vertical structure of subtropical evergreen broadleaf forest communities using UAV LiDAR technology, effectively extracting canopy height and tree location information (Xie et al., 2020). Xu et al. used UAV imagery and LiDAR point clouds to estimate forest stand characteristic variables in subtropical natural secondary forests, while Yuan used airborne LiDAR technology to estimate the accumulation models of four typical coniferous forests (Korean pine, Larch, Red pine, and Spruce) in the Northeast forest region, finding that point cloud height variables contributed the most to biomass models (Xu et al., 2015; Yuan et al., 2021). These studies indicate that LiDAR-derived feature variables can estimate and invert forest structure parameters at the stand scale, but the optimal biomass model varies with the scope and purpose of the survey.

Recent studies integrating LiDAR technology with biomass estimation of *Cunninghamia lanceolata* have primarily focused on optimal single-tree segmentation, height growth prediction, and carbon storage estimation. The structural parameters of Chinese fir extracted using airborne LiDAR generally correspond well with known growth patterns, highlighting the practical value of this approach (Yu et al., 2023; Zhou et al., 2023; Yu et al., 2024). However, current research on Chinese fir biomass remains relatively limited. Existing models often neglect critical factors such as stand age and site conditions, which can introduce significant bias into biomass estimates. Furthermore, most available models concentrate on total tree biomass, with limited attention given to individual tree components (Guo et al., 2022). To improve model precision and applicability, it is essential to account for varying stand developmental stages and to incorporate a broader range of variables that capture both intra- and inter-group variation, thereby better elucidating the influence of stand structure on biomass accumulation. This study addresses these gaps by focusing on Chinese fir plantations across eight regions in Guangdong Province. Utilizing high-density UAV LiDAR point cloud data, the research extracts 57 forest structural variables—including height, intensity, canopy cover, and leaf area index. The objectives are threefold: (1) to assess the predictive utility of elevation, density, and intensity metrics derived from LiDAR data; (2) to construct age-group-specific biomass models for different tree components; and (3) to evaluate and compare the accuracy of various modeling approaches to identify the most effective estimation strategy. By systematically analyzing the influence of stand developmental stages on biomass modeling for *Cunninghamia lanceolata*, this study aims to enhance estimation accuracy and provide a scientifically rigorous framework for large-scale forest carbon stock assessment, precision forestry, and sustainable ecosystem management.

## 2 Materials and methods

### 2.1 Study area

**Figure 1:**
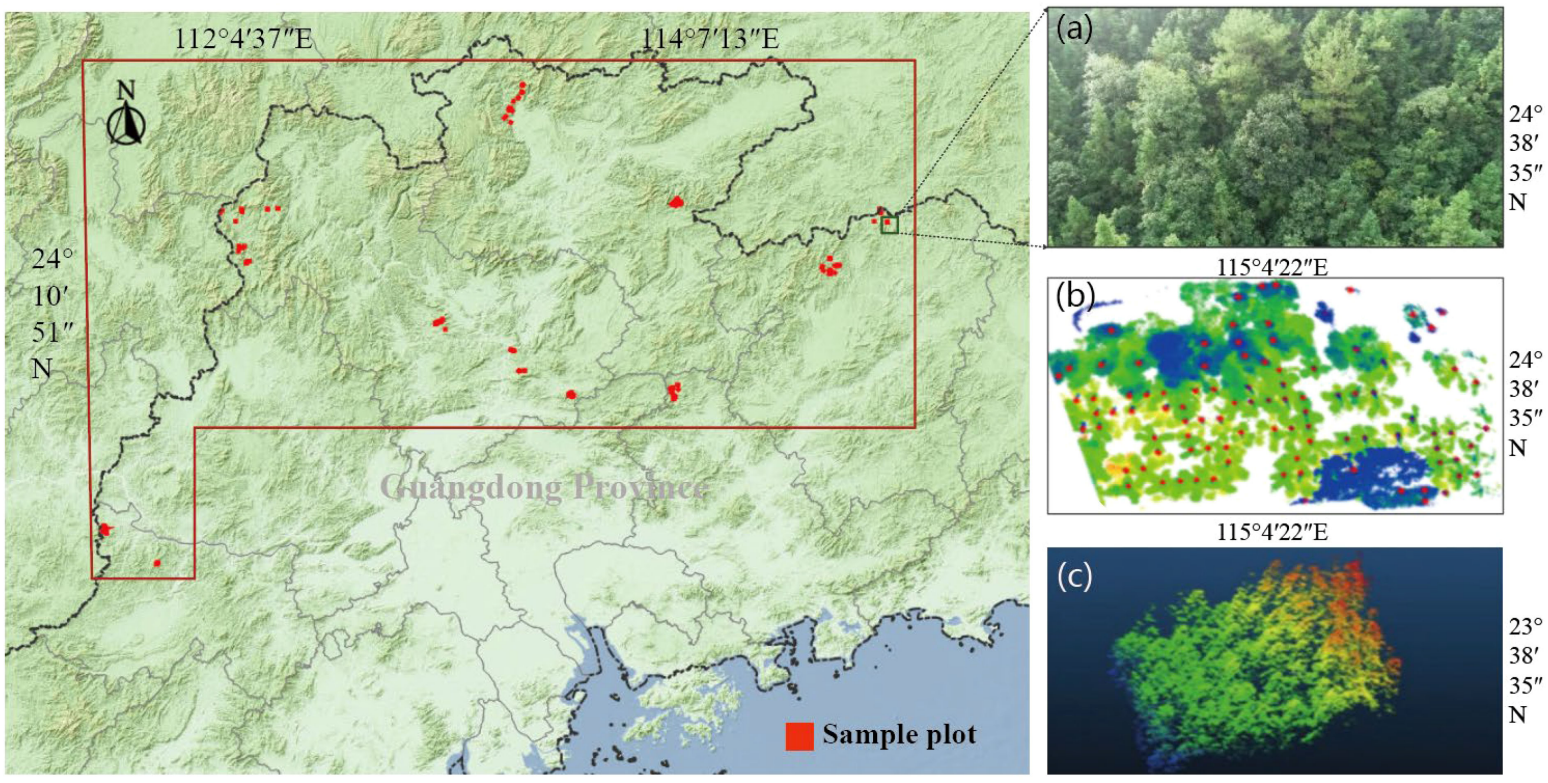
Location of study area and sample distributions across North-central Guangdong, China (subfigures: **(a)** orthorectified RGB image of the sample plot, **(b)** single-tree segmentation output derived from canopy analysis, and **(c)** LiDAR point cloud map of the sample plot).

The study area is located in the northern and central parts of the Nanling Mountains in Guangdong Province, China. The geographical coordinates range from 23°2′12″N to 25°17′29″N and 111°22′55″E to 115°4′54″E (**Figure 1**). This region is a major distribution area of Chinese fir, where the species has been cultivated for many years. The presence of well-established stands across various age groups provides an ideal setting for research and development. The terrain is generally higher in the north and lower in the south, with mountains and high hills in the north and plains and terraces in the south. The elevation ranges from 53 m to 580 m, and the annual rainfall is between 1300 mm and 2500 mm, belonging to the East Asian subtropical monsoon climate zone (Huang et al., 2022). Sample plots were set up in state-owned forest farms in eight counties (cities) within the region. The soil is mountainous red soil with a slightly acidic pH value and is primarily sandy loam with a thickness of about 50 cm. The main tree species in the forest farms include Cunninghamia lanceolata, Pinus massoniana, and Eucalyptus spp., with pure plantations being predominant.

### 2.2 Data sources

#### 2.2.1 Sample plots

In September 2023, following a comprehensive reconnaissance of the study area, 154 sample plots of 30 m × 30 m were selected based on forest type, stand age, and management practices. This study considered 33 plots of young forests, 34 of middle-aged forests, 26 of near-mature forests, 34 of mature forests, and 27 of over-mature forests. Real-time kinematic (RTK) GPS was used to obtain the geographical coordinates and elevation data for each tree and plot center. Terrain factors such as topography, slope, and altitude were recorded. All trees with a diameter at breast height (DBH) ≥ 5 cm in the sample plots were measured for tree species, DBH, height, height to the first branch, crown width, and canopy density. DBH was measured using a measuring tape (at a height of 1.3 m from the ground). Total tree height and height to the first branch were measured using a TruPuls 360 laser rangefinder; crown width was measured in two perpendicular directions (east-west and north-south).

Forest biomass estimation based on the tree measurement factors includes above-ground biomass, under-ground biomass, wood biomass, bark biomass, branch biomass, and leaf biomass. above-ground biomass includes all wood biomass and non-wood biomass (leaf, branch, bark, leaf). The allometric growth equation was used to calculate the above-ground biomass and its components in the sample plots (Zeng, 2013; Xiang et al., 2021; Sun et al., 2023). It can be expressed as (**Equation 1**):


(1) $$\left\{ \begin{array}{l} M_{Above-ground}=a_{1}*D^{b_{1}}*H^{c_{1}}\\ M_{Trunk}=\frac{1}{1+g_{1}+g_{2}+g_{3}}*M_{Above-ground}\\ M_{Bark}=\frac{g_{1}}{1+g_{1}+g_{2}+g_{3}}*M_{Above-ground}\\ M_{Branch}=\frac{g_{2}}{1+g_{1}+g_{2}+g_{3}}*M_{Above-ground}\\ M_{Leaf}=\frac{g_{3}}{1+g_{1}+g_{2}+g_{3}}*M_{Above-ground}\\ M_{Under-ground}=a_{2}*D^{b_{2}}*H^{c_{2}} \end{array}\right.$$

In the formula, $M_{Trunk}$ , $M_{Bark}$ , $M_{Branch}$ , and $\;M_{Leaf}$ represent the biomass of stand trunk, bark, branches, and leaves components, respectively, measured in kilograms (kg). $M_{Above-ground}$ and $M_{Under-ground}$ are the estimated values of stand aboveground biomass, and underground biomass, while $g_{1}$ , $g_{2}$ , and $g_{3}$ are the ratio functions of bark, branches, and leaves relative to wood biomass, which is set to 1. According to the established standards, the specific calculation formulas are as follows:

$\;g_{1}=0.37301H^{-0.29282},\;$ $g_{2}=0.80058D^{0.79098}H^{-1.29690},\;$ $g_{3}=3.23395D^{0.48038}H^{-1.71324}$ ,

where D is the average diameter at breast height of the forest stand and H is the average tree height of the forest stand.

#### 2.2.2 Lidar data

UAV-based LiDAR data was acquired in the study area. The flight platform used was a quadcopter drone equipped with the Huace AS-1300HL LiDAR system, operating at a wavelength of 1550 nm with an accuracy of 5 mm. The system had a scanning angle of 330°, an effective scanning field of ±30°, a laser beam divergence of 0.5 mrad, a pulse duration of 3.5 ns, and a side overlap greater than 50%, achieving a point density of approximately 110 points/m². The LiDAR data underwent preprocessing using the commercial software LiDAR360, where it was normalized and classified to generate the Digital Surface Model (DSM), Digital Elevation Model (DEM), and Canopy Height Model (CHM) (Zhang et al., 2016).

Vertical distribution variables of LiDAR point clouds are commonly used for estimating forest biomass. These variables can quantitatively describe the height distribution of vegetation canopies, such as height percentiles, density percentiles, and height statistics (Holmgren, 2004; Næsset and Gobakken, 2005; Zhang et al., 2016). The Leaf Area Index (LAI), which is one of the most fundamental parameters characterizing canopy structure, is defined as half of the total leaf surface area per unit ground area and is calculated from the normalized vegetation points in the point cloud data (White et al., 2015). The point cloud data was denoised and filtered to obtain noise points, ground points, and vegetation points. The classified vegetation points were then normalized for elevation to eliminate the effects of terrain, resulting in a normalized point cloud (Yan et al., 2020). Based on the vector boundaries of sample plots, the normalized point cloud for each plot was clipped, and 57-point cloud feature variables were extracted, including height percentiles, cumulative height percentiles, height statistics, and density percentiles (**Table 1**).

**Table 1:** Description of LiDAR metrics.

Parameter type	Parameter name	Description
Canopy characteristics	Leaf area index (LAI)	Half the surface area of all leaves per unit surface area
	Degree of coverage (C_C_)	Ratio of the number of vegetation points in the first Echo to the number of vegetation points in the first echo
Height percentile	*h_1_*, *h_5_*, *h*_10_, …, *h*_80_, *h*_90_, *h*_95_, *h*_99_	Where are the points at X% height within the normalized LiDAR point cloud
Cumulative height percentile	*AIH*_1_, *AIH_5_*, *AIH*_10_, …, *AIH*_80_, *AIH*_90_, *AIH*_95_, *AIH*_99_	Cumulative height of points located at X% height within normalized LiDAR point cloud
Height statistic	*h*_max_	Maximum Z value of all points
	*h*_mean_	Average Z value of all points
	*h*_cv_	Coefficient of variation of point cloud height
	*h*_sd_	The standard deviation of Z value of all points in a statistical unit
	*h*_skewness_	Skewness value of point cloud height
	*h*_kurtosis_	kurtosis of point cloud height
Intension percentiles	*d*_1_, *d*_2_, *d*_3_, …, *d*_7_, *d*_8_, *d*_9_	Intensity percentile of the point cloud

### 2.3 Model construction

Based on field-measured data of Chinese fir plots and corresponding LiDAR data, univariate and bivariate models were constructed for different stand biomass categories. The optimal biomass model was selected through model comparison, and age groups were further incorporated to develop an inversion model.

#### 2.3.1 Basic model

This study, limited by the available measured sample data, utilized RStudio software to establish biomass estimation models for Chinese fir. A total of 154 samples were selected as the dataset for model development. Widely used biomass models, including linear, logarithmic, exponential, and power functions, were chosen as the basic models to examine the relationship between dependent and independent variables (Andersen et al., 2006; Trofymow et al., 2014) (**Table 2**). To address the issue of reduced degrees of freedom due to an excessive number of independent variables, a correlation test was used to select variables. To avoid multicollinearity within the models, only variables that significantly contributed to stand biomass were selected. The initial analysis was conducted by fitting the models with the entire dataset, and the optimal basic model was selected based on model evaluation metrics (Chen et al., 2022).

**Table 2:** Basic model form to describe forest biomass variations.

No.	Model	Model source
I	AGB = b_0_ + b_1_ X_1_ + b_2_ X_2_ + b_3_ X_3_ + … + b_n_ X_n_ + ${\text{ϵ}}_{AGB}$	Linear
II	AGB = b_0_/[1 + b_1_ exp(- b_2_ X_1_ - b_3_ X_2_ - b_4_ X_3_ - … - b_n + 1_ X_n_)] + ${\text{e}}_{AGB}$	Logistic
III	AGB = b_0_ exp(- b_1_ X_1_ - b_2_ X_2_ - b_3_ X_3_ - … - b_n_ X_n_) + ${\text{ϵ}}_{AGB}$	Exponential
IV	AGB = $b_{0}X_{1}^{b_{1}}X_{2}^{b_{2}}X_{3}^{b_{3}}\cdot \cdot \cdot X_{n}^{b_{n}}$ + ${\text{ϵ}}_{AGB}$	Empirical

In the model parameters, AGB is the aboveground biomass calculated by allometric growth equation (Eq. 1), X_1_,X_2_, X_3_, …, X_n_ are the height variable and intensity variable extracted from lidar LiDAR, b_0_, b_1_, b_2_, …,b_n_ are the parameter to be estimated, ${\text{ϵ}}_{AGB}$ represents the error term in each equation.

#### 2.3.2 Nonlinear mixed-effects model

A mixed-effects model was constructed based on the linear and nonlinear relationships between the regression function and both fixed effects and random effects parameters (Pinheiro and Bates, 2000). The general form of a single-level mixed-effects model is as follows (**Equation 2**):


(2) $$\{\begin{array}{c} Y_{ij}=f\left(\theta_{ij},x_{ij}\right)+\epsilon_{ij}\;\;\\ \theta_{ij}=\theta A_{ij}\beta +B_{ij}u_{i}\\ \epsilon_{ij}∼N(0,\;\sigma^{2}),u_{i}∼N(0,\;D)\\ u_{i}=1,\ldots ,M;j=1,\ldots ,M \end{array}$$

where: $x_{ij}$ and $Y_{ij}$ represent the explanatory variable and response variable for the *j* plot in the *i* group, respectively; $\theta_{ij}$ is the parameter vector; $\epsilon_{ij}\;$ is the error term, assumed to follow a normal distribution; $f\left(\theta_{ij},x_{ij}\right)$ is the aboveground biomass model; $A_{ij}$ and $B_{ij}$ are the design matrices of *β* and $u_{i}$ , respectively; β is the fixed effects parameter vector; $u_{i}$ represents the random effect generated by the *i* group; ϵ and u are independent of each other; *D* is the covariance matrix of the random effects.

#### 2.2.3 Dummy variable model

A dummy variable model was introduced to describe the impact of different stand development stages on biomass. All Chinese fir stands in the study area were classified into five stages: young forest, middle-aged forest, near-mature forest, mature forest, and over-mature forest. Corresponding variables $\;Q_{i}$ =( $Q_{1}$ , $Q_{2}$ , $Q_{3}$ , $Q_{4}$ , $Q_{5}$ )were set to represent these stages. In this approach, variables are assigned values to represent qualitative or categorical data. $Q_{1}$ =(1, 0, 0, 0) represents young forest; $Q_{2}$ =(0, 1, 0, 0)represents middle-aged forest; $Q_{3}$ =(0, 0, 1, 0) represents near-mature forest; $Q_{4}$ =(0, 0, 0, 1) represents mature forest; $Q_{5}$ =(0, 0, 0, 0) represents over-mature forest.

The significance of parameters that include dummy variables was tested using the t-test. If the dummy variable was not significant at a level of α=0.05, it was excluded, and the model was refitted. This process was repeated until all parameters would be significant (Dong et al., 2023).

#### 2.2.4 Random forest

The Random Forest model uses the bootstrap sampling to construct multiple decision trees for regression prediction. The final prediction is generated by aggregating the results of these trees, typically by averaging the predictions for regression problems or taking the mode for classification problems (Dinh et al., 2016; Deng et al., 2023). The feature vector calculation formula for each decision tree in the Random Forest is as follows (**Equation 3**):


(3) $$\left\{ \begin{array}{l} min_{j,m}\left[\sum_{q_{i}\in R_{1}\left(j,m\right)}{\left(q_{i}-\hat{q_{R_{1}}}\right)}^{2}+\sum_{q_{i}\in R_{2}\left(j,m\right)}{\left(q_{i}-\hat{q_{R_{2}}}\right)}^{2}\right]\\ \;\hat{q_{R_{1}}}=mean\left[\;q_{i}|q_{i}\in R_{1}\left(j,m\right)\right]\\ \;\hat{q_{R_{2}}}=mean\left[\;q_{i}|q_{i}\in R_{2}\left(j,m\right)\right] \end{array}\right.$$

where: j and m are the feature vectors of the decision tree, respectively; $R_{1}\left(j,m\right)$ is the set of samples of the first child node obtained by splitting feature m on node j, $R_{2}\left(j,m\right)$ is the sample set of the second child node obtained after splitting feature m on node j; $q_{i}$ is the actual measured value of the i-th sample; $\hat{q_{R_{1}}}\;$ and $\hat{q_{R_{2}}}\;$ are the predicted values of stand biomass for $R_{1}\left(j,m\right)$ and $R_{2}\left(j,m\right)$ , respectively. The study chose to import the sample biomass data and LiDAR variables for simulation with the help of Random forest function in RStudio to derive the predictions.

### 2.4 Model accuracy evaluation criteria

The fitting performance of the models was evaluated using coefficient of determination (R²), relative residual, root mean square error (RMSE), and total relative error (TRE) (Fu et al., 2018; Guo et al., 2022). We randomly divided the data into two datasets, with 70% of the data used for model training (108 sample plots) and the other 30% for model validation (46 sample plots).

To further assess the model, verification set covering different age groups were used to obtain the predicted biomass values for these test samples. A linear regression relationship was then established between the predicted values and the observed values, expressed a as y=b+ax, with b corresponding coefficients and R² values. When a is close to 1, b is close to 0, and R^2^ is high, it indicates minimal bias between predicted and observed values, signifying high prediction accuracy of the model.


(4) $$\bar{e}=\sum^{}e_{i}/n=\frac{\sum_{i=1}^{n}\left(y_{i}-\hat{y_{i}}\right)}{n}$$

(5) $$\sigma^{2}=\sum_{i=1}^{n}{\left(e_{i}-\bar{e}\right)}^{2}/\left(n-1\right)$$

(6) $$R^{2}=1-\frac{\sum_{i=1}^{n}{\left(y-\hat{y}\right)}^{2}}{\sum_{i=1}^{n}{\left(y-\bar{y}\right)}^{2}}$$

(7) $$\;TRE=\frac{\sum_{i=1}^{n}{\left(y-\hat{y}\right)}^{2}}{\sum_{i=1}^{n}y^{2}}*100$$

(8) $$RMSE=\sqrt{{\bar{e}}^{2}+\sigma^{2}}$$

(9) AIC=2k−lnl

where $\;y_{i}$ and $\hat{y_{i}}$ are the stand biomasses estimated by the allometric equation and predicted by the newly developed biomass model, respectively, and y is the mean biomass by the allometric equation; and n is the number of sample plots; and k is the number of model parameters; and l is the likelihood function value; and $\bar{e}$ , $\sigma^{2}$ , $R^{2}$ , TRE and RMSE are the mean bias, variance of bias, coefficient of determination, total relative error and root mean square error, respectively. RMSE is defined as the combination of the mean bias and its variance and is the most important evaluation criterion of the model.

## 3 Results and analysis

### 3.1 Statistical results of measured data

Tree height and diameter at breast height (DBH) were measured for 33,628 Chinese fir trees within the sample plots. The results showed that the number of Chinese firs per plot ranged from 39 to 478, with a canopy density between 0.35 and 0.90. The average DBH (calculated using the quadratic mean method) ranged from 6.10 cm to 28.24 cm, and the average tree height ranged from 5.05 m to 21.35 m. The slope of the plots varied between 6°and 37°, with most plots located on eastern slopes. Based on the measured tree height and DBH, the aboveground biomass in the plots was calculated using an allometric growth equation, resulting in a range of 41.92 t/hm² to 523.90 t/hm², with an average value of 228.90 t/hm². Detailed statistical data are presented in **Table 3**.

**Table 3:** Biomass characteristics of all age groups for modeling.

Dataset	Age group	Sample plot number	Statistics	Crown density	Stand density (trees/hm²)	Mean DBH (cm)	Mean tree height(m)	Mean branch height(m)	Basal area (m²/hm²)	Trunk (t/hm^2^)	Bark(t/hm^2^)	Branch(t/hm^2^)	Leaf (t/hm^2^)	Above-ground biomass(t/hm^2^)	Below-ground biomass(t/hm^2^)
Training set	young	23	Min.	0.45	2100	6.1	5.0	1.9	12.18	24.81	4.20	1.40	4.39	34.81	7.11
			Max.	0.90	4167	15.3	11.8	6.3	50.33	158.63	25.90	19.45	24.17	227.32	35.00
			Mean.	0.71	3242	12.2	9.3	4.9	37.81	105.86	17.32	12.06	16.28	151.51	23.76
	middle-aged	24	Min.	0.35	844	9.2	6.9	3.4	15.32	39.64	6.45	4.90	5.95	57.04	8.52
			Max.	0.90	4355	22.8	16.7	10.1	60.16	186.95	30.42	28.96	25.81	269.18	40.32
			Mean.	0.66	3065	13.2	10.5	6.3	39.96	121.71	19.82	15.54	18.35	175.41	26.40
	near-mature	18	Min.	0.60	1611	11.0	6.4	3.8	30.35	76.31	12.55	7.99	12.00	108.85	17.84
			Max.	0.90	3711	17.1	14.5	8.9	57.92	206.75	33.29	31.99	29.79	301.82	41.38
			Mean.	0.75	2563	14.5	11.5	6.4	40.78	136.25	22.03	20.07	19.98	198.32	28.16
	mature	24	Min.	0.40	1078	13.3	8.6	3.9	29.64	76.30	12.44	9.38	11.59	109.71	16.77
			Max.	0.90	2478	26.2	21.3	12.6	71.68	307.66	48.58	72.37	41.08	469.58	54.32
			Mean.	0.66	1764	19.2	13.8	7.4	19.30	179.29	28.57	35.80	24.78	268.44	33.45
	over-mature	19	Min.	0.40	822	13.4	8.8	4.2	30.41	85.63	13.91	11.54	12.80	123.88	17.18
			Max.	0.90	2856	26.9	20.0	10.8	54.07	216.54	34.36	53.86	29.46	329.48	39.40
			Mean.	0.68	1767	17.3	12.9	6.9	39.01	144.42	23.08	27.43	20.22	215.15	27.57
Validation set	young	10	Min.	0.70	2878	9.1	6.3	2.1	25.96	60.20	10.01	4.94	9.89	85.05	15.16
			Max.	0.90	5311	13.0	10.3	5.7	40.00	124.73	20.35	16.57	19.02	180.67	27.65
			Mean.	0.80	3543	11.4	8.6	4.1	35.49	100.93	16.56	11.02	15.71	144.21	23.13
	middle-aged	10	Min.	0.40	867	10.2	9.1	5.0	18.72	50.85	8.32	5.70	7.80	72.70	11.41
			Max.	0.85	4355	22.8	16.7	8.3	49.20	204.40	32.98	30.56	29.70	297.65	41.54
			Mean.	0.64	2448	14.6	11.9	6.9	35.15	126.37	20.50	17.21	18.77	182.85	26.70
	near-mature	8	Min.	0.60	1267	12.5	9.8	5.4	34.50	114.92	18.70	14.66	17.30	165.57	24.29
			Max.	0.85	2811	18.8	14.3	7.1	43.06	145.56	23.46	24.24	21.27	213.84	29.94
			Mean.	0.73	1911	16.6	12.7	6.2	39.25	134.67	21.71	20.66	19.49	196.53	27.19
	mature	10	Min.	0.60	911	14.2	11.4	4.9	18.60	128.46	20.66	20.77	18.40	118.29	25.38
			Max.	0.90	3244	23.1	18.4	8.0	23.90	220.70	35.04	37.61	27.51	331.74	39.98
			Mean.	0.80	1836	18.2	14.1	6.7	21.50	170.97	27.29	32.62	23.78	254.65	32.25
	over-mature	8	Min.	0.40	433	19.5	14.1	6.8	27.14	101.26	15.93	25.84	13.26	156.29	17.18
			Max.	0.80	1400	28.2	20.0	9.4	42.62	186.01	28.83	40.62	25.12	281.22	33.28
			Mean.	0.66	1047	21.9	16.9	8.4	37.46	153.04	24.29	32.57	20.78	230.69	27.66

**Figure 2:**
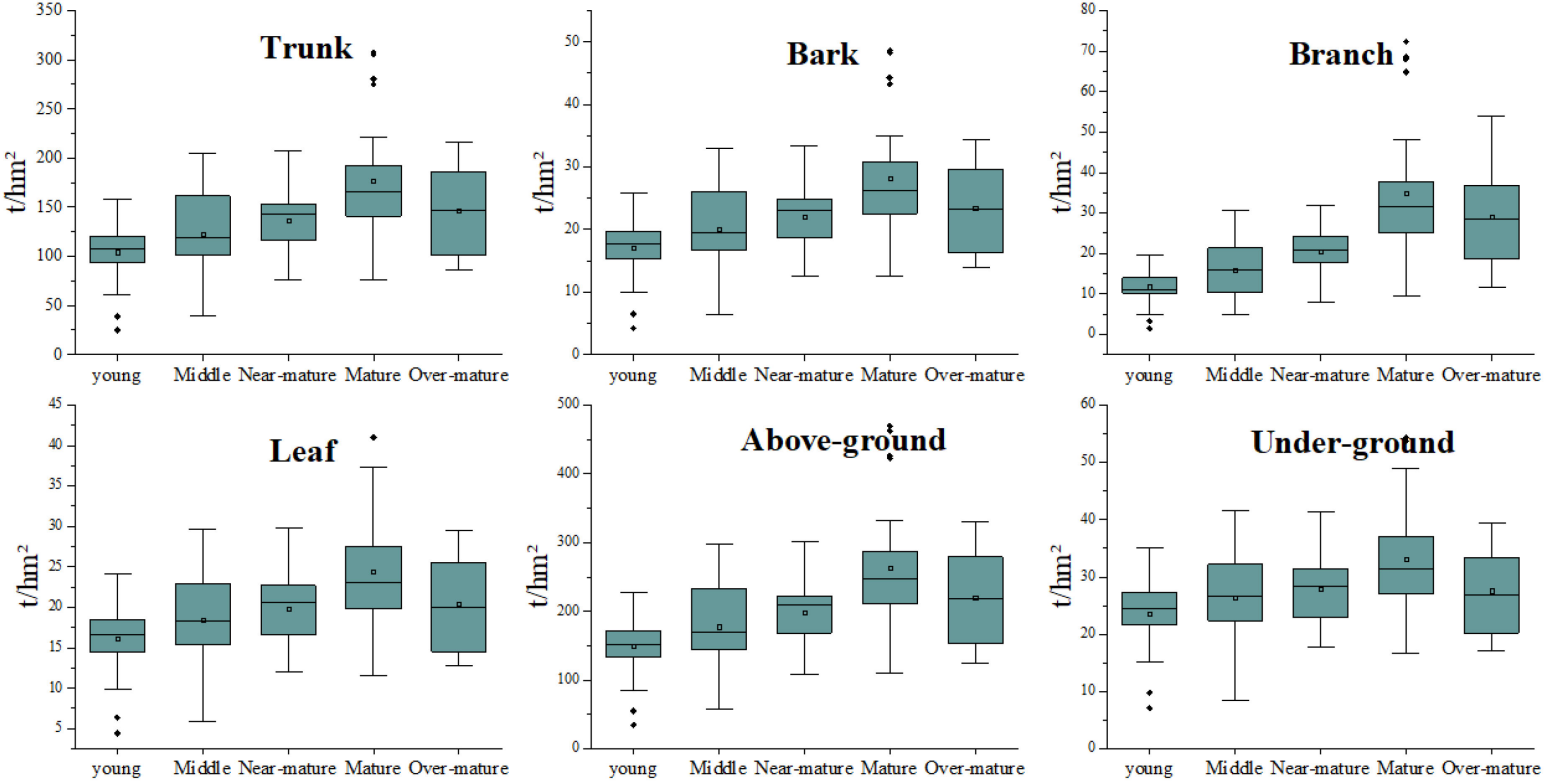
Boxplots of the six measured stand biomass for different forest types (The subfigures represent the biomass distribution across different tree components in the following order: Trunk, bark, branches, leaves, aboveground, and underground across various age groups).

There are differences in parameters such as tree height and biomass across different age groups of Chinese fir. As shown in **Figure 2**, the biomass of young, middle-aged, near-mature, and mature forests shows an upward trend, while the biomass of over-mature forests slightly decreases compared to mature forests. In terms of dispersion, the biomass distribution within different plots is relatively concentrated for young, near-mature, and mature forests, while it is more scattered among over-mature forests. Regarding outliers, young forests tend to have more instances of extremely low biomass values, while mature forests have more instances of relatively high biomass values.

### 3.2 Correlation analysis of feature variables

A Pearson correlation analysis was conducted between LiDAR point cloud variables and plot biomass, revealing 41 significantly correlated feature variables. The biomass of the trunk, bark, aboveground, and belowground components showed a high correlation with LiDAR point cloud variables, with Pearson correlation coefficients generally above 0.4. The correlation between branch and leaf biomass and LiDAR point cloud variables was moderate. Specifically, leaf biomass had a significant correlation with only seven LiDAR point cloud variables (P<0.05). The intensity variables had a generally low correlation with the measured biomass, with an average correlation coefficient of only 0.445.

**Figure 3:**
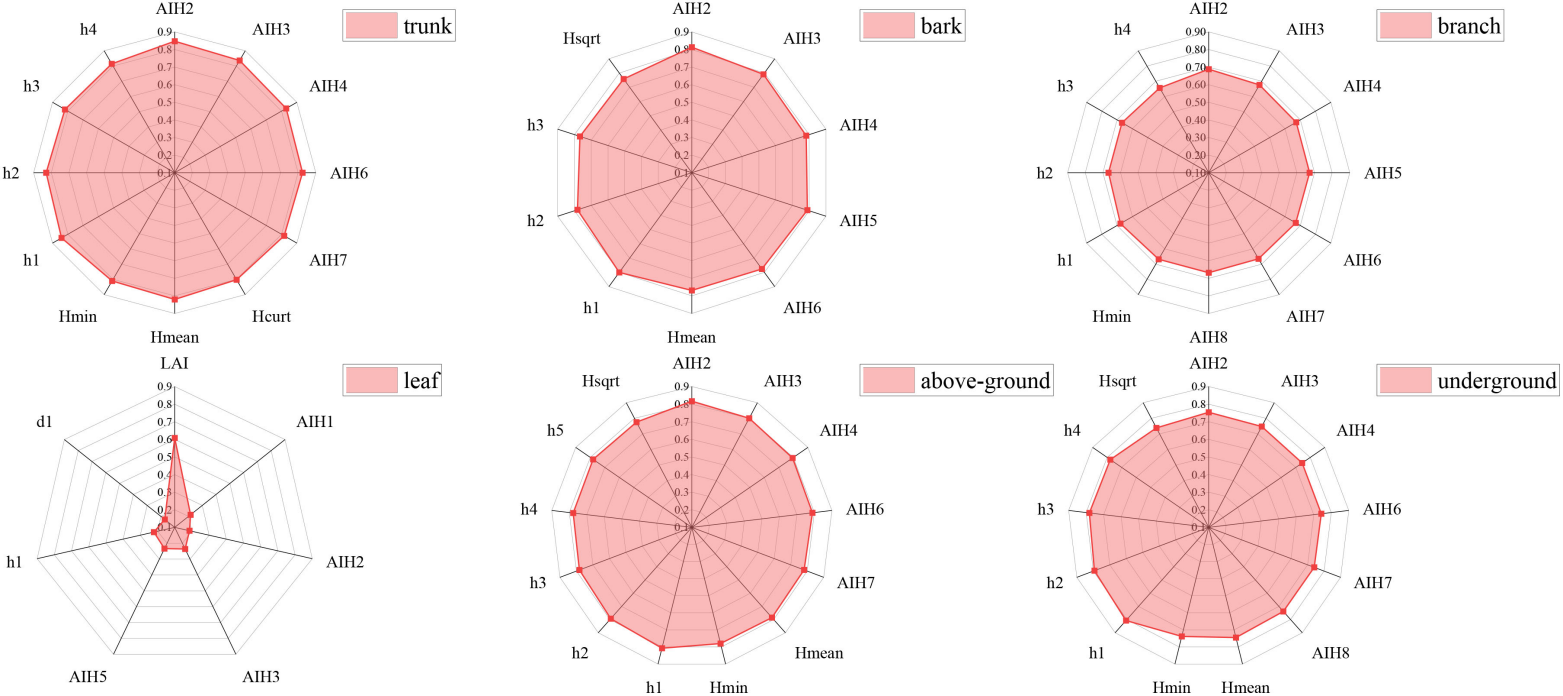
Pearson’s correlation coefficient between the selected metric and measured biomass (AIH1, AIH2,…, and LAI represent the corresponding LiDAR characteristic variables in sequential order, as detailed in **Table 1**).

Variables with a high correlation to different biomass components of the stand were selected. As shown in **Figure 3**, height percentiles (h1, h2) and cumulative height percentiles (AIH2, AIH3, AIH4, AIH6, AIH7) exhibited high Pearson correlation coefficients with various biomass components. Notably, the 5% cumulative height percentile had the highest correlation coefficients with trunk biomass (0.847), bark biomass (0.813), branch biomass (0.688), and aboveground biomass (0.817). Leaf biomass had the highest correlation with the Leaf Area Index (LAI), with a correlation coefficient of 0.608.

### 3.3 Model construction and accuracy analysis

#### 3.3.1 Base model

To enhance model accuracy, variance inflation factor (VIF<10) analysis was used to remove multicollinearity among the variables. These were then analyzed for correlation with various biomass components of the forest stands. After screening, only three LiDAR feature variables—cumulative height percentile AIH2, Leaf Area Index (LAI), and intensity percentile d9—had a VIF of less than 10. Based on the correlation analysis results in the previous text, the 5% cumulative height percentile AIH2 and LAI were chosen as the parameters for the equations. The estimation accuracy results of the univariate and bivariate regression base models are presented in **Tables 4**, **5**. Due to space constraints, only the validation set results are presented in the article.

**Table 4:** Statistical indicators for the validation set of the univariate biomass model based on stand factors.

Indicator	Logistic						Linear					
	Trunk	Bark	Branch	Leaf	Above- ground	Under- ground	Trunk	Bark	Branch	Leaf	Above- ground	Under- ground
**RMSE**	2.167	2.167	3.479	1.332	19.289	4.367	13.307	2.144	3.269	1.468	19.427	4.337
**R^2^**	0.588	0.588	0.269	0.560	0.621	0.495	0.686	0.597	0.355	0.466	0.616	0.502
**TRE**	3.068	3.068	3.392	1.764	2.875	2.991	3.308	3.001	3.030	2.151	2.904	2.950
**AIC**	437.273	437.273	531.670	334.510	867.247	591.068	793.626	439.081	538.136	358.860	869.107	594.088
**Variable**	AIH_2_	AIH_2_	AIH_2_	LAI	AIH_2_	AIH_2_	AIH_2_	AIH_2_	AIH_2_	LAI	AIH_2_	AIH_2_

Indicator	Exponential						Empirical					
	Trunk	Bark	Branch	Leaf	Above- ground	Under- ground	Trunk	Bark	Branch	Leaf	Above- ground	Under- ground
**RMSE**	13.383	2.157	3.296	1.562	19.634	4.392	12.737	2.091	3.184	1.413	18.672	4.177
**R^2^**	0.682	0.592	0.344	0.422	0.607	0.490	0.712	0.617	0.388	0.504	0.645	0.538
**TRE**	3.341	3.025	3.058	2.331	2.956	3.009	3.073	2.883	2.891	1.191	2.711	2.759
**AIC**	793.586	437.181	533.944	366.381	867.967	591.789	799.36	446.377	542.413	351.594	874.883	598.527
**Variable**	AIH_2_	AIH_2_	AIH_2_	LAI	AIH_2_	AIH_2_	AIH_2_	AIH_2_	AIH_2_	LAI	AIH_2_	AIH_2_

The bolded values represent the biomass of different components of Chinese fir, with the following correspondences: 'Trunk' (trunk biomass), 'Branch' (branch biomass), 'Bark' (bark biomass), 'Leaf' (leaf biomass), 'Above ground' (aboveground biomass), and 'Under ground' (underground biomass).

**Table 5:** Statistical indicators for the validation set of the bivariate parameter biomass model based on stand factors.

Indicator	Logistic						Linear					
	Trunk	Bark	Branch	Leaf	Above- ground	Under- ground	Trunk	Bark	Branch	Leaf	Above- ground	Under- ground
**RMSE**	1.852	1.852	3.024	1.327	16.471	3.813	12.197	1.928	2.939	1.450	17.456	3.906
**R^2^**	0.700	0.700	0.448	0.564	0.724	0.615	0.736	0.674	0.479	0.479	0.690	0.596
**TRE**	2.319	2.319	2.646	1.750	2.165	2.345	2.840	2.490	2.513	2.098	2.403	2.450
**AIC**	416.633	416.633	519.453	335.821	850.536	580.622	787.018	421.944	526.336	361.073	856.661	585.710

Indicator	Exponential						Empirical					
	Trunk	Bark	Branch	Leaf	Above- ground	Under- ground	Trunk	Bark	Branch	Leaf	Above- ground	Under- ground
**RMSE**	12.151	1.950	3.039	1.509	17.782	4.034	11.134	1.800	2.801	1.401	16.080	3.667
**R^2^**	0.738	0.667	0.443	0.436	0.678	0.570	0.780	0.716	0.527	0.514	0.737	0.644
**TRE**	2.841	2.554	2.675	2.275	2.500	2.608	2.424	2.206	2.299	1.954	2.074	2.184
**AIC**	782.482	417.547	523.401	368.673	853.529	583.87	787.185	426.498	532.259	354.362	859.607	590.205

The bolded values represent the biomass of different components of Chinese fir, with the following correspondences: 'Trunk' (trunk biomass), 'Branch' (branch biomass), 'Bark' (bark biomass), 'Leaf' (leaf biomass), 'Above ground' (aboveground biomass), and 'Under ground' (underground biomass).

From the model evaluation results, in the univariate regression model, the 5% cumulative height percentile AIH2 as an independent variable showed a significantly better fit than LAI. Moreover, only leaf biomass selected LAI as a parameter in the optimal model. Considering the accuracy evaluation of both regression models, the R² values for the univariate regression model ranged from 0.269 to 0.712, with a median of 0.537. In contrast, the R² values for the bivariate regression model ranged from 0.443 to 0.780, with a median of 0.619, and most were above 0.6. When comparing RMSE, TRE, and AIC values, the bivariate regression model also reflected a better fit. Therefore, the bivariate regression model better explains the variations in biomass among the different components of Chinese fir stands, and this analysis focuses primarily on the bivariate regression model.

The fitting results indicate that UAV LiDAR point cloud feature variables have a good fitting effect on trunk, branch, aboveground, and belowground biomass, with most R² values above 0.6. The fitting effect for branch and leaf biomass was relatively poor, with leaf biomass showing the worst fit, having R² values around 0.5. This suggests the need to introduce more parameters to improve the fitting model for leaf biomass.

Comparing the four base models, the results show that the Empirical model had the highest R² values and the lowest RMSE and TRE values for predicting the biomass of the trunk, branches, bark, aboveground, and belowground components, indicating high predictive accuracy for the biomass of Chinese fir stands. For leaf biomass prediction, the Logistic model had a higher R² value, and its TRE value of 1.750 was the lowest among all models, suggesting that this model provides a better fit for leaf biomass and has high predictive accuracy. Additionally, the AIC values of the Logistic model were all at low levels.

Based on the model accuracy results, the following models were selected as the base models for predicting the biomass of each component (**Equation 10**).


(10) $$\left\{ \begin{array}{l} B_{Trunk}=a_{0}H_{2}{\;}^{a_{1}}LAI^{a_{2}}+{\text{ ϵ}}_{SG}\\ B_{Bark}=b_{0}H_{2}{\;}^{b_{1}}LAI^{b_{2}}+{\text{ ϵ}}_{SP}\\ B_{Branch}=c_{0}H_{2}{\;}^{c_{1}}LAI^{c_{2}}+{\text{ ϵ}}_{SZ}\\ B_{Leaf}=\;d_{0}\;/\;\left[1\;+\;d_{1}\text{ exp}\left(\;-\;d_{2}{\text{ H}}_{2}\;-\;d_{3}\;LAI\;\right)\right]\;+{\text{ ϵ}}_{SY}\\ B_{Above-ground}=e_{0}H_{2}{\;}^{e_{1}}LAI^{e_{2}}+{\text{ ϵ}}_{DS}\\ B_{Under-ground}=f_{0}H_{2}{\;}^{f_{1}}LAI^{f_{2}}+{\text{ ϵ}}_{DX} \end{array}\right.$$

#### 3.3.2 Nonlinear mixed-effects models

**Figure 4:**
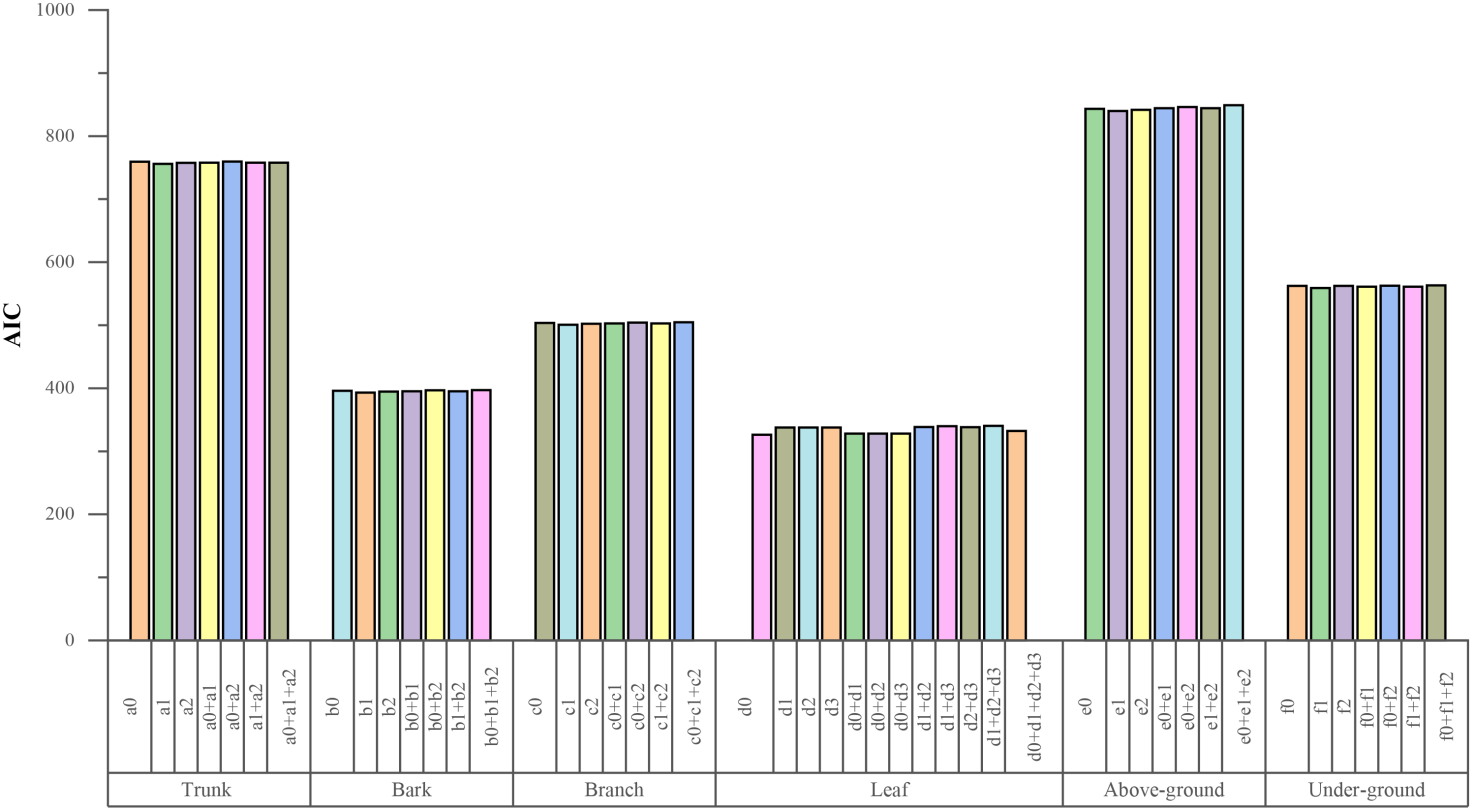
AIC values of non-linear random effects combination for each sub item biomass (a0, a1, …f2 refer to the parameters needs to be included as random effects; + refer to the combinations of the random effects).

To increase the accuracy of the base models, nonlinear mixed effects models (NLME) were constructed in this study. Considering the effects of different management practices, various age group were treated as random variables influencing the parameters a0~a2、b0~b2、… 、f0~ f2​, resulting in a total of 50 NLME models. Out of these, 47 models were statistically significant, with AIC values ranging from 326.222 to 848.969 (**Figure 4**). The results showed that the AIC values within the model groups were relatively similar, but there were substantial differences between groups.

The following NLME models for Chinese fir biomass components exhibited optimal fitting statistics. Parameter estimates, standard errors, adjusted R², RMSE, and TRE are shown in **Table 6**. For the fitted models, the adjusted R² values for each biomass component model were above 0.65. Simultaneously, the RMSE and TRE values decreased, reflecting an improved fit. At a significance level of 0.05, all parameters in the NLME models were statistically significant, indicating that the explanatory variables had a notable impact on the biomass of various forest components.

**Table 6:** Fitting results of nonlinear mixed effects model for various tree component biomass.

Component		Estimate (SE)				Training set			Verification set		
		$\beta_{i_{0}}$	$\beta_{i_{1}}$	$\beta_{i_{2}}$	$\beta_{i_{3}}$	RMSE	R^2^	TRE	RMSE	R^2^	TRE
Trunk	NLME	6.4926 (1.176)	0.8025 (0.0646)	0.3231 (0.0773)		10.696	0.832	2.107	10.238	0.814	2.002
	Dummy	6.184 (1.040)	0.8325 (0.0650)	0.3118 (0.0664)		10.897	0.815	2.690	11.996	0.798	2.236
Bark	NLME	1.5321 (0.2319)	0.6430 (0.0556)	0.3483 (0.0666)		1.670	0.826	1.806	1.686	0.751	1.895
	Dummy	1.4815 (0.2126)	0.6825 (0.0564)	0.3181 (0.0582)		1.805	0.808	2.168	1.864	0.678	2.362
Branch	NLME	4.0062 (0.6072)	0.3927 (0.0557)	0.3642 (0.0718)		2.730	0.739	2.102	2.799	0.559	2.121
	Dummy	4.5820 (0.6515)	0.3735 (0.0586)	0.3097 (0.0623)		2.996	0.672	2.589	2.863	0.657	2.255
Leaf	NLME	11.2872 (0.4570)	9.0153 (4.0861)	0.0175 (0.0306)	0.7148 (0.1378)	1.146	0.674	1.302	1.327	0.563	1.762
	Dummy	12.7999 (1.3018)	2.4059 (0.6328)	0.0589 (0.0353)	0.2875 (0.0934)	1.433	0.569	1.875	1.405	0.549	1.856
Above-ground	NLME	12.6607 (2.0266)	0.6715 (0.0535)	0.3724 (0.0668)		15.496	0.831	1.830	15.233	0.763	1.825
	Dummy	14.3207 (2.1072)	0.6657 (0.0580)	0.3092 (0.0601)		17.104	0.804	2.294	16.445	0.796	1.927
Under-ground	NLME	3.5641 (0.6163)	0.5960 (0.0584)	0.3386 (0.0732)		3.841	0.761	2.296	3.507	0.674	1.974
	Dummy	4.9122 (0.7274)	0.4795 (0.0600)	0.2994 (0.0634)		4.043	0.732	2.609	3.994	0.705	1.737

In **Table 6**, “NLME” represents the nonlinear mixed-effects model, while “Dummy” denotes the dummy variable model.

#### 3.3.3 Dummy variable models

Compared to the NLME models, the dummy variable models showed a reduction in R², with increases in RMSE and TRE values, indicating slightly poorer fit for the biomass of Chinese fir (**Table 6**).When compared to the corresponding univariate and bivariate models, the fit of the biomass models improved, with noticeable enhancements in branch biomass model (Training set R^2^ = 0.672, RMSE=2.996; Verification set R^2^ = 0.657, RMSE=2.863), while the leaf biomass fitting did not show significant changes.

**Figure 5:**
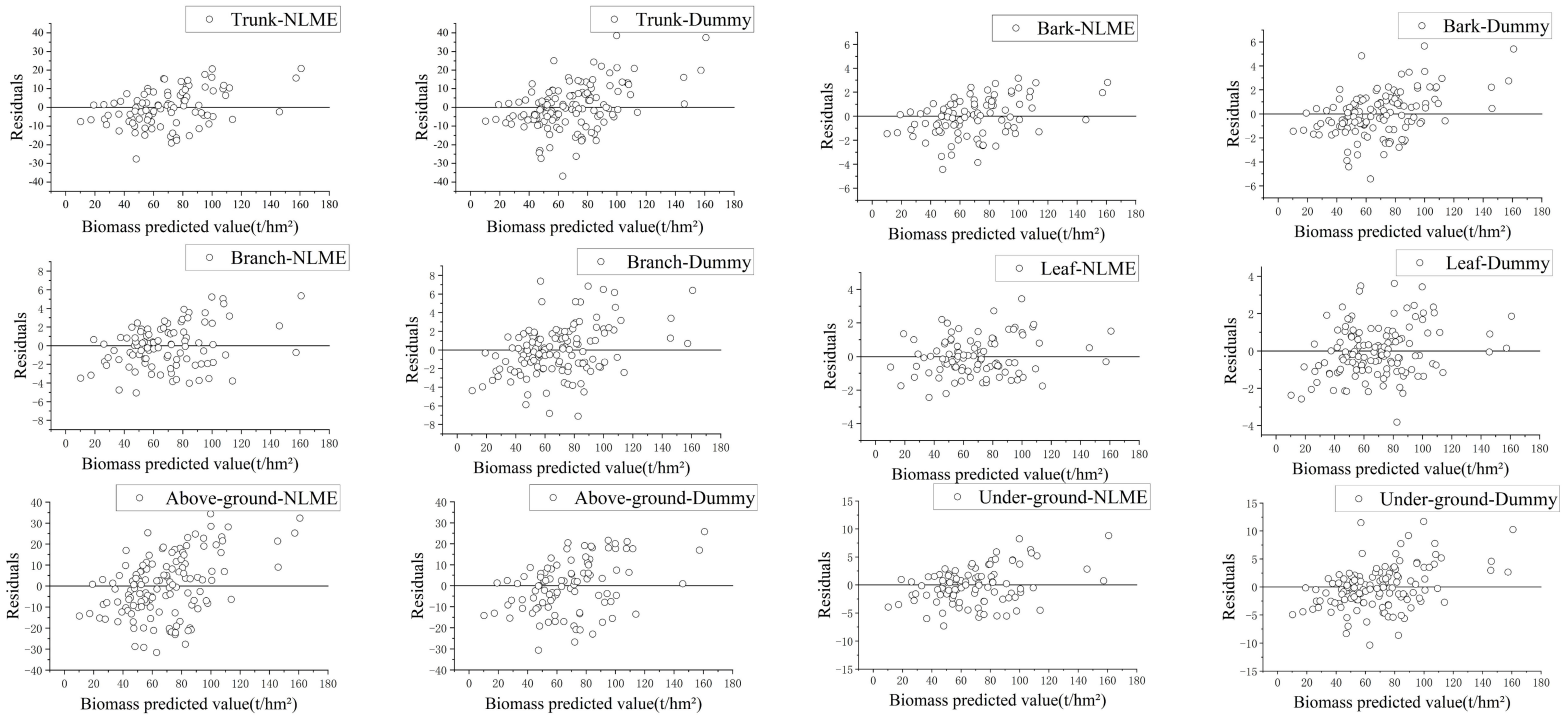
Residual distribution diagram of nonlinear mixed effects model and dummy variable model (“-NLME” represents the residual of the nonlinear mixed-effects model for each branch, “-Dummy” represents the residual of the dummy variable model for each branch).

**Figure 5** shows the residual plots for the NLME and dummy variable models showed that residuals are uniformly distributed around zero, with no apparent trends of divergence or convergence related to predicted biomass values. This suggests that there are no significant systematic biases or heteroscedasticity, and the NLME model residuals are closer to zero with fewer outliers.

#### 3.3.4 Machine learning methods

Compared to traditional parametric models, the Random Forest algorithm demonstrated certain advantages (**Table 7**). The R^2^ value has increased compared to the average of the base model, random effects model, and dummy variable model, demonstrating a significant improvement in fitting accuracy. The largest improvements were seen in leaf biomass (Training set R^2^ = 0.855, RMSE=0.804; Verification set R^2^ = 0.862, RMSE=0.934), where the parameter models had lower fitting accuracy.

**Table 7:** Fit statistics of machine learning models.

Component	Training set			Verification set		
	RMSE	R^2^	TRE	RMSE	R^2^	TRE
Trunk	7.223	0.939	0.095	6.163	0.910	0.150
Bark	1.080	0.935	0.082	1.113	0.880	0.127
Branch	1.758	0.900	0.074	1.705	0.855	0.106
Leaf	0.804	0.855	0.066	0.934	0.862	0.074
Above-ground	9.902	0.937	0.081	9.400	0.894	0.119
Under-ground	2.277	0.920	0.076	2.246	0.857	0.117

### 3.4 Model validation

**Figure 6:**
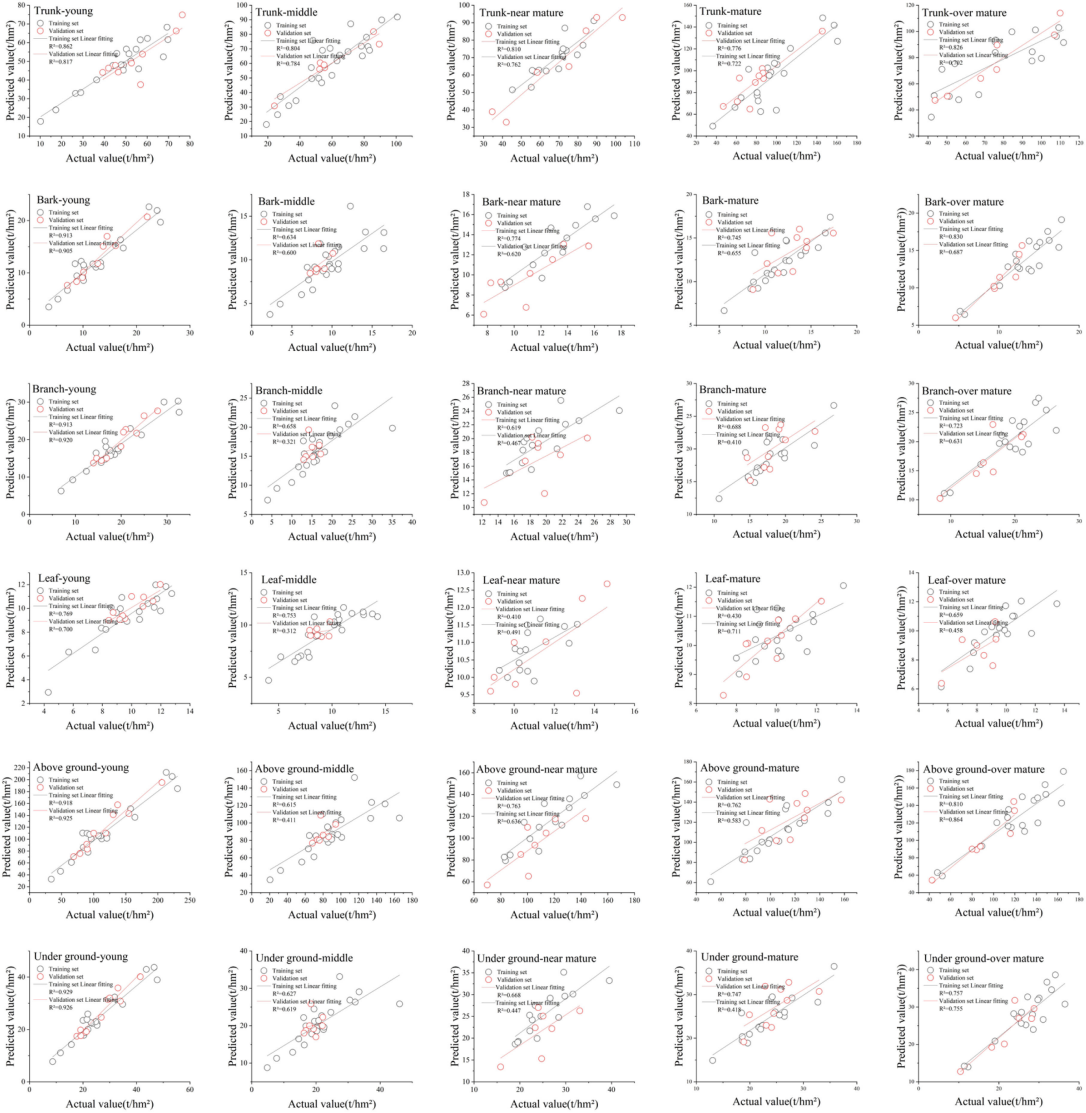
Fitting diagram of estimated and observed values of nonlinear mixed effects model (Trunk, Bark, Branch, Leaf, Aboveground, and Underground represent the biomass distribution of different components of Chinese fir, while Young, Middle, Near-Mature, Mature, and Over-Mature indicate the respective age groups).

Due to the more reasonable distribution of relative residuals in NLME, this study employed the Logistic and Empirical models, incorporating age groups as mixed effects, the validation sample data were then use in the NLME models to obtain predicted value. The calculations show that the majority of *a* values fall within the range of 0.6 to 1.1, while most *b* values remain between -1 and 5, despite a few extreme values exceeding 10. Overall, the scatter plot of the model shows a balanced distribution around the fitting line. Across different age groups, the model performs better for young and middle-aged forests, while the fitting accuracy for near-mature forests is relatively lower compared to the other four age groups. In terms of different biomass components, except for leaf biomass, there is no significant deviation between the observed and estimated values for other components of Chinese fir biomass. Notably, the estimation accuracy for trunk biomass and aboveground biomass is higher than that of other components. **Figure 6** displays the fit between the predicted and observed values for the NLME models.

The diagnostic analyses in **Figures 5**, **6** and **Table 7** the demonstrate that the model in this study exhibits a good overall fit, with no significant heteroscedasticity. The bivariate models provided a better fit for biomass than the univariate models, and tree height was found to be a significant factor influencing the models. The inclusion of age group factors in the NLME and dummy variable models led to improvements over conventional models, while machine learning methods significantly enhanced the fitting accuracy over the NLME model.

## 4 Discussion

This study constructed biomass inversion models for *Cunninghamia lanceolata* (Chinese fir) stands using UAV-based LiDAR point cloud data integrated with field survey measurements. The parametric and non-parametric models achieved coefficients of determination of R² = 0.832 and R² = 0.939, respectively, indicating high predictive accuracy. These results are comparable to those reported by Yu et al. (2023) and outperform several previously developed UAV-LiDAR-based biomass estimation models for Chinese fir (Yu et al., 2023; Xian et al., 2024). This demonstrates the reliability and applicability of LiDAR-derived structural metrics in estimating stand biomass, providing critical methodological support for biomass estimation and carbon stock assessments at the regional scale.

Remote sensing-based biomass modeling, particularly using LiDAR, offers significant advantages in characterizing vertical forest structure and spatial heterogeneity, addressing limitations inherent in traditional ground-based inventory methods. However, challenges remain regarding optimal variable selection, model transferability, and reduced accuracy in structurally complex or heterogeneous forest stands. Model accuracy is not only contingent upon stand structure and geographic characteristics but is also influenced by the specific LiDAR-derived metrics selected for modeling (Li et al., 2019; Chen et al., 2022). In this study, a comprehensive set of LiDAR metrics was extracted, including 46 height variables, 9 intensity variables, and 2 density variables. Following comparative analysis, two key variables—cumulative 5th percentile height and Leaf Area Index (LAI)—were selected as final predictors. These findings are consistent with previous research by Du et al. (2021) and Qu et al. (2014), who identified percentile-based height metrics and canopy cover as dominant variables in biomass modeling (Qu et al., 2014; Du et al., 2021). Height-related metrics were found to effectively capture mean stand height, while density and intensity variables reflected canopy closure and horizontal structure. The integration of these three types of features enhances the capability of LiDAR-based models to accurately estimate biomass across various stand conditions (Wallace et al., 2016).

Regarding model architecture, four commonly used base models were tested, with enhancements including nonlinear mixed-effects modeling, dummy variables, and machine learning techniques. Model performance was assessed using R², RMSE, TRE, and residual diagnostics (Li et al., 2022). Results revealed that different base models were optimal for different biomass components: the Logistic model was best suited for foliage biomass, while the Empirical model provided superior performance for other components. Overall, nonlinear models demonstrated better fit and stability than linear regressions, aligning with previous findings (Fu et al., 2018; Chen et al., 2022).

The inclusion of stand age as a covariate in the mixed-effects models significantly improved model accuracy, indicating that developmental stage plays a critical role in biomass accumulation. This aligns with the work of Guo (2022), who highlighted the influence of stand age in ground-based biomass models for Chinese fir (Zhao et al., 2020). In managed plantations—such as those in Guangdong Province where initial planting densities are high—tending and thinning practices during early to mid-rotation stages alter stand density and structural attributes (e.g., DBH, height, volume), thereby impacting biomass distribution (Jiang et al., 2022). Furthermore, both dummy-variable models and mixed-effects models were evaluated. The dummy-variable models showed slightly lower accuracy compared to nonlinear mixed-effects models, which may be attributed to the selection of input variables and sample size distribution among age classes. This aligns with the guidance of Wang (2008) and Chen (2018), who suggest that when the number of categories is small (<10), dummy-variable models are suitable, whereas mixed-effects models are more appropriate when category count is higher or sample sizes are imbalanced (Wang et al., 2008; Chen et al., 2017).

Component-wise biomass modeling showed the following order of model performance: trunk > aboveground > bark > underground > branch > leaf. This ranking is consistent with the results reported by Sun et al. (2021), who also found trunk and underground biomass to be more predictable than foliage biomass (Sun et al., 2023). As stands mature, increases in DBH and height lead to higher trunk, bark, and underground biomass, while branch and leaf biomass are more sensitive to stand density and light competition. Including stand age in foliage biomass models improved their accuracy, though performance remained lower than for other components. This may explain why the foliage biomass model required a distinct base function (Logistic) to accommodate its nonlinear growth pattern.

Despite the strong performance of the proposed models, several limitations remain. While stand age was incorporated, other environmental factors such as site conditions and canopy closure were not included and may have significant impacts on biomass allocation. Future research could incorporate two-level or multi-level mixed-effects models to account for these sources of variability. Moreover, this study focused on monospecific plantations, and further investigations in mixed-species forests are necessary to explore the effects of species composition and structural complexity. Prior studies have indicated that LiDAR detection accuracy may be reduced in steep terrain or multi-layered canopies, warranting methodological refinement in such contexts (Wang et al., 2008; Khosravipour et al., 2015; Chen et al., 2017; Jiang et al., 2022). Additionally, only the Random Forest algorithm was evaluated among non-parametric methods. Future work should include other machine learning approaches, such as Support Vector Machines (SVM), Artificial Neural Networks (ANN), and K-Nearest Neighbor (KNN), to compare their performance against traditional models. Due to space limitations, integrated estimation of component and total biomass will be discussed in a follow-up paper.

## 5 Conclusion

This study focused on Chinese fir plantations in Guangdong Province and employed a combination of airborne LiDAR data and ground-measured data to construct biomass models for different components of forest stands using both parametric and non-parametric modeling methods. Forest stand age was incorporated as a random factor in the models. The following conclusions were drawn based on the fitting and validation results:

1. Multicollinearity among LiDAR-derived variables: There is significant multicollinearity among the characteristic variables derived from UAV LiDAR data, with height variables showing a strong positive correlation with stand biomass. The 5% cumulative height percentile and leaf area index (LAI) were identified as more suitable predictor variables.
2. Good fitting performance of basic models: Both univariate and bivariate basic models demonstrated good fitting performance, with coefficients of determination R^2^ generally above 0.6. The fitting accuracy was highest for trunk and branch biomass, while leaf biomass model showed relatively lower fitting accuracy.
3. Impact of stand development stage on biomass: The stand developmental stage significantly influences the biomass of Chinese fir stands, making it essential to consider stand age when constructing stand models. Nonlinear mixed-effects models demonstrated higher fitting accuracy compared to dummy variable models. Machine learning methods significantly improved model fitting accuracy.
4. High predictive accuracy of the models: The models developed in this study exhibited high predictive accuracy, with all errors remaining within reasonable limits. These models are suitable for estimating the biomass of Chinese fir stands at the scale of Guangdong Province and can be applied in practical scenarios.

## Data Availability

The raw data supporting the conclusions of this article will be made available by the authors, without undue reservation.
